# Quantitative assessment of individual populations within polymicrobial biofilms

**DOI:** 10.1038/s41598-018-27497-9

**Published:** 2018-06-22

**Authors:** Susana Patrícia Lopes, Nuno Filipe Azevedo, Maria Olívia Pereira

**Affiliations:** 1 Centre of Biological Engineering, LIBRO – Laboratório de Investigação em Biofilmes Rosário Oliveira, University of Minho, Campus de Gualtar, 4710-057 Braga, Portugal; 2 LEPABE – Dep. of Chemical Engineering, Faculty of Engineering, University of Porto, Rua Dr. Roberto Frias, s/n, 4200-465 Porto, Portugal

## Abstract

Selecting appropriate tools providing reliable quantitative measures of individual populations in biofilms is critical as we now recognize their true polymicrobial and heterogeneous nature. Here, plate count, quantitative real-time polymerase chain reaction (q-PCR) and peptide nucleic acid probe-fluorescence *in situ* hybridization (PNA-FISH) were employed to quantitate cystic fibrosis multispecies biofilms. Growth of *Pseudomonas aeruginosa*, *Inquilinus limosus* and *Dolosigranulum pigrum* was assessed in dual- and triple-species consortia under oxygen and antibiotic stress. Quantification methods, that were previously optimized and validated in planktonic consortia, were not always in agreement when applied in multispecies biofilms. Discrepancies in culture and molecular outcomes were observed, particularly for triple-species consortia and antibiotic-stressed biofilms. Some differences were observed, such as the higher bacterial counts obtained by q-PCR and/or PNA-FISH (≤4 log_10_ cells/cm^2^) compared to culture. But the discrepancies between PNA-FISH and q-PCR data (eg *D*. *pigrum* limited assessment by q-PCR) demonstrate the effect of biofilm heterogeneity in method’s reliability. As the heterogeneity in biofilms is a reflection of a myriad of variables, tailoring an accurate picture of communities´ changes is crucial. This work demonstrates that at least two, but preferentially three, quantification techniques are required to obtain reliable measures and take comprehensive analysis of polymicrobial biofilm-associated infections.

## Introduction

In most natural scenarios, including in infectious diseases, microorganisms assemble in dynamic communities and persist within high spatially structured consortia, known as biofilms^1,2^. Such living structures display unique properties, providing strong benefits to their constituent species (e.g. enhanced resistance to antimicrobial therapy, protection towards host immunity, better adaptation to hostile surrounding conditions)^3–5^. The recognition that most biofilms present a spatiotemporal heterogeneous chemical, physiological and genetic composition^6,7^ and typically comprise multiple species^8^ poses a serious concern in health care regarding the synergies that arise from the residing species that generally turn infections more severe and recalcitrant to treatment^5,9,10^. This highlights the need for reliable technologies that comprehensively diagnose polymicrobial biofilm infections, by clearly addressing each individual member in the community, for accurate and timely therapeutic decisions.

Traditional diagnosis of biofilm-associated infections has relied on culture-based approaches to identify the aetiological agents, as well as to ascertain for the most abundant members^11–14^. Conventional techniques are, however, time-consuming and frequently lead to false-negative results, for numerous reasons: they require appropriate selective media, microbiological techniques and optimal growth conditions for an accurate detection/identification; antibiotic-treated bacteria are, in most cases, below the detection limit of culture^12^; “viable but nonculturable” (VBNC) bacteria are often evaded from detection, since a great percentage (>70%) of microorganisms inhabiting human body surfaces are not readily cultured *in vitro*^15–17^. Importantly, bacteria encased in biofilms are notoriously difficult to culture or even grow poorly on agar plates^18^. Under these circumstances, standard microbiological methods are often ill-suited to diagnose polymicrobial biofilm infections^19^ and underscore the reason that clinicians struggle to manage these infections with culture-independent tools^20–23^. Quantitative real-time polymerase chain reaction (q-PCR) has been the “gold standard” in clinic, affording a rapid screening and quantification of specific organisms in biofilm samples that are unable to be detected by cultivation^24–26^. Fluorescence *in situ* Hybridization (FISH) using peptide nucleic acid (PNA) probes (i.e. PNA-FISH) has also been evidenced as an attractive molecular tool with regard to a rapid identification of medically relevant species in a variety of polymicrobial contexts^27–33^. Rapid technological advances hold promises, however the multiple bacteria residing in a biofilm, typically possessing distinct behaviours, phenotypes, physiological/metabolic states, might compromise the reliability of molecular methods in biofilms^34–38^. While the mechanisms underpinning the level of heterogeneity generated in the biofilm – which is in a large extent a reflection of a myriad of variables (e.g. antibiotic administration^39^; the physicochemical characteristics of the local microenvironment^7,40^) - are not completely exploited, selecting appropriate tools that give robust measures of the community changes has potential clinical significance for opportunities for therapeutic breakthroughs.

This work aims to employ and compare culture (plate count) and molecular (q-PCR and PNA-FISH) approaches to quantitatively assess individual populations in mixed-species biofilms. As a case-study, a defined polymicrobial consortia involving phylogenetically diverse bacterial strains related with cystic fibrosis (CF) infections were used. Specifically, *Pseudomonas aeruginosa* was assessed, in two- and triple-species biofilms, with the CF lesser common species *Inquilinus limosus* and *Dolosigranulum pigrum*, under environments with distinct oxygen availabilities (aerobic, AER; microaeropilic, MAER; and anaerobic, ANAER) and following antibiotic intervention.

## Results and Discussion

**Figure 1:**
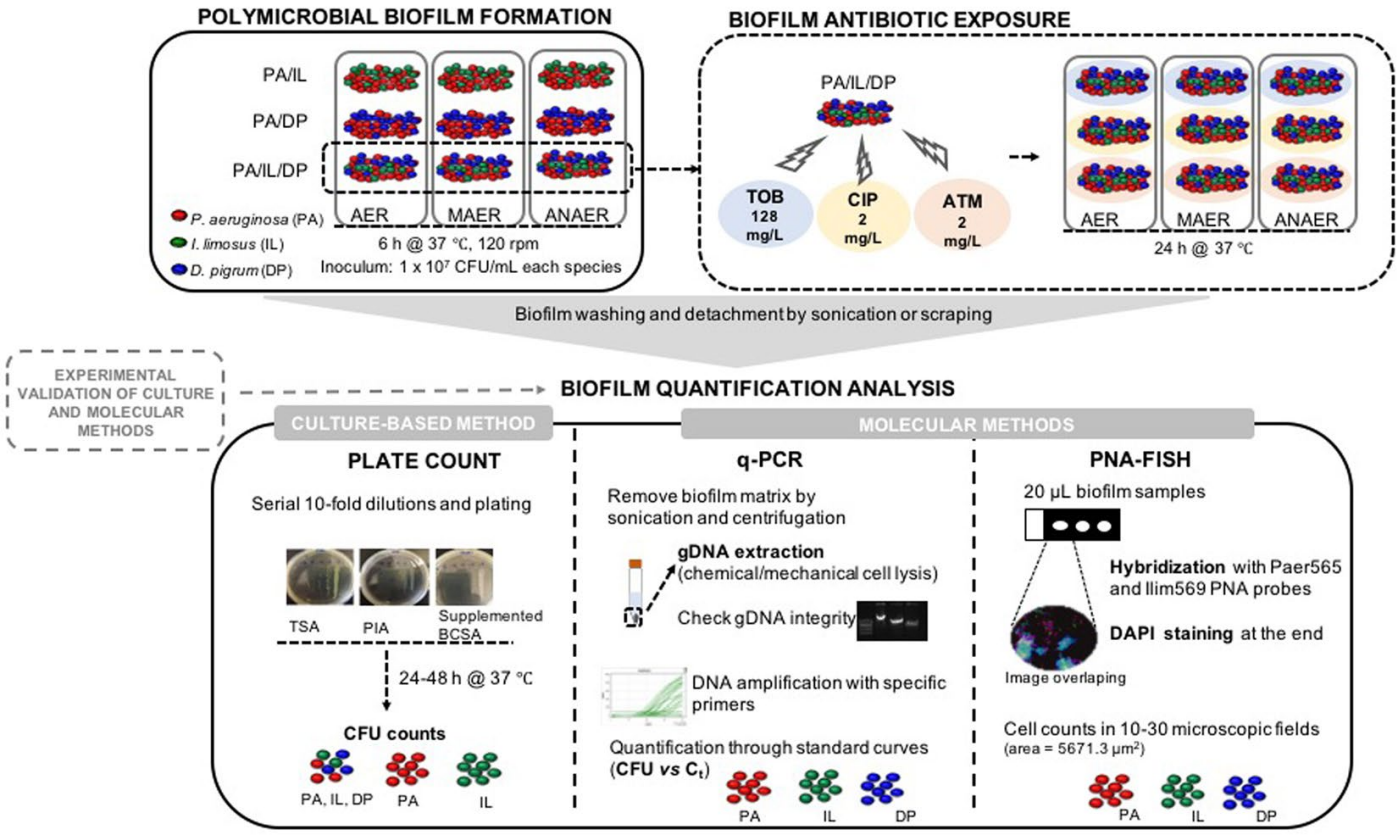
Experimental design and workflow. Two- and triple-species biofilms involving *P*. *aeruginosa*, *I*. *limosus*, and *D*. *pigrum* developed under aerobic, microaerophilic, and anaerobic environments and the triple consortia exposed to antibiotics were assessed through culture (plate count) and molecular (q-PCR and PNA-FISH) methods. In culture-based method, individual biofilm populations were quantified by unspecific and selective growth media. Regarding q-PCR, DNA extracted from the biofilm-cells was amplified by using specific designed oligonucleotide primers and individual biofilm populations quantified by previous established standard curves (plotting CFU/mL vs *C*_*t*_ for pure cultures). A multiplex PNA-FISH assay with an additional staining step with DAPI was performed *ex situ*, using specific PNA probes - Paer565 and Ilim569 - previously developed and optimized^46^ and bacteria were then estimated using an epifluorescence microscope. Experimental validation was performed for each technique before biofilm quantification experiments. Abbreviations: PA = *P*. *aeruginosa*, IL = *I*. *limosus*, DP = *D*. *pigrum*, AER = aerobic, MAER = microaerophilic, ANAER = anaerobic, TOB = tobramycin, CIP = ciprofloxacin, ATM = aztreonam.

CF lung has long been pointed out as the major site of infection mediated by multispecies biofilms. For many years, culture-based diagnostic microbiology has been the mainstay of CF clinical care^11^, however underestimating the presence of biofilms in these infections. The remarkable advances achieved with molecular approaches in the last decades has led to an improvement in the CF diagnosis, by often detecting previously undescribed levels of microbial diversity^20–22,41^. In parallel to the array of microbes, complex local microenvironments (containing gradients of nutritional/carbon sources and regions with a range of oxygen potentials) invariably exist in the CF airways^42^. Through our progressive understanding of the complexity of the CF milieu, it becomes increasingly apparent that the CF sociomicrobiology (i.e. how microbes assemble, how they function and how they change in the community) is shaped by a myriad of factors acting collectively^7,40,43^. Detailed and robust measures of these changes in biofilm communities are urgently required, and become even more critical if the activity (and possibly, the further selection) of a given antibiotic treatment is dictated by the composition of the community, as has been recently denoted^40,44,45^. Using phylogenetically distinct species identified in CF samples^46^, we defined three consortia that could apparently be representative of the CF infections, encompassing the prominent pathogen *P*. *aeruginosa* and two lesser common species *I*. *limosus* (a gram-negative aerobe) and *D*. *pigrum* (gram-positive, facultative anaerobe)^40,46^. Such populations were quantitatively assessed through culture and molecular techniques in biofilms challenged by environments with variable oxygen and antibiotic treatment. The experimental design and workflow of our strategy is shown in Fig. 1.

### Experimental validation of culture, q-PCR and PNA-FISH methods in planktonic populations

**Figure 2:**
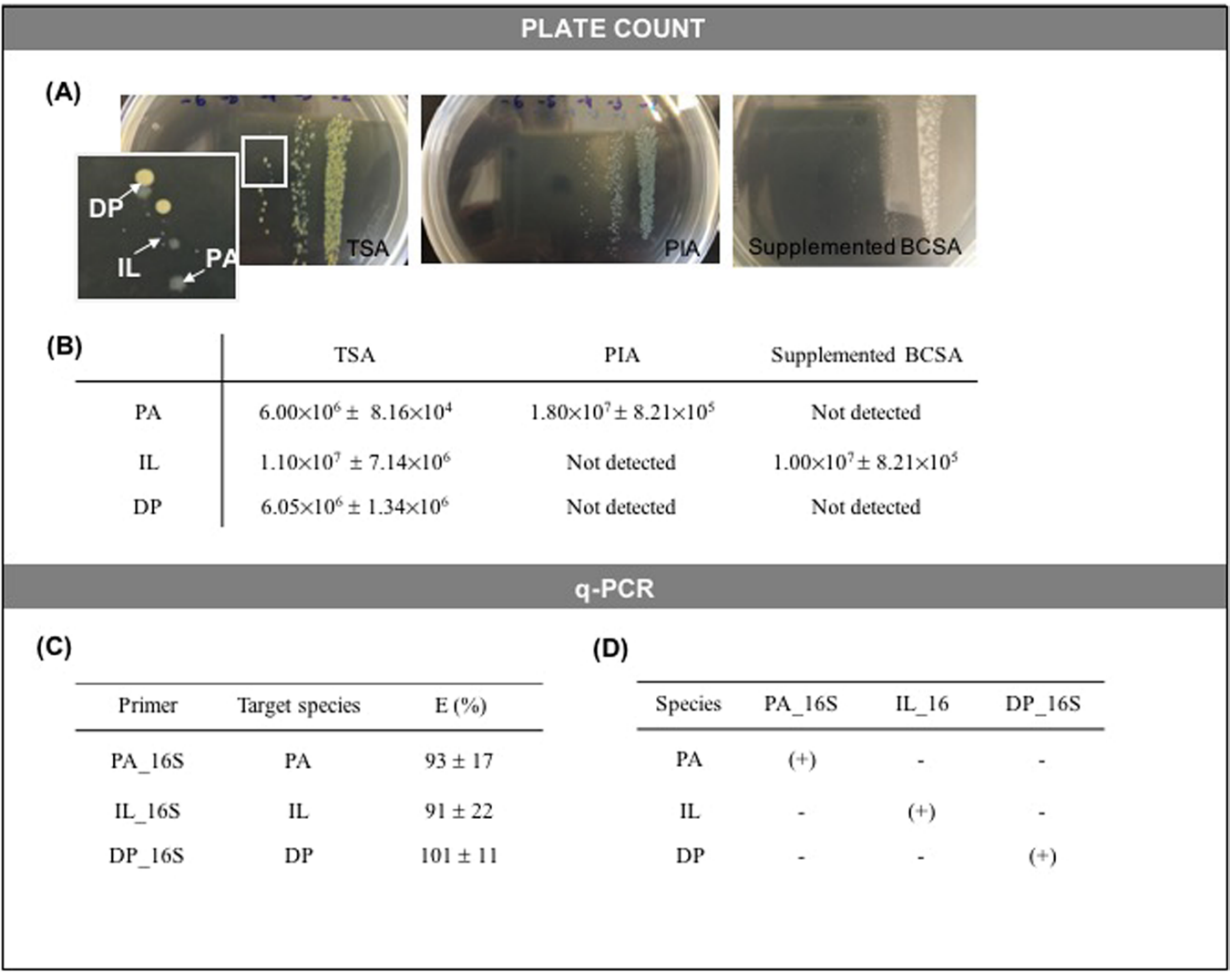
Validation of culture and q-PCR methods. (**A**) Discrimination of *P*. *aeruginosa*, *I*. *limosus* and *D*. *pigrum* colonies on unspecific (TSA) and on specific culture medium (PIA and supplemented BCSA). PIA was specific for *P*. *aeruginosa* and BCSA supplemented with 100 mg/L ticarcillin and 300 000 IU/L polymyxin B was specific for *I*. *limosus* after extended incubation time; (**B**) Bacterial counts (expressed as means ± standard deviations (SDs) CFU/mL for three independent assays) obtained on TSA on specific selective media. Bacterial counts were monitored on solid growth media after incubation at 37 °C for 24–48 h from a mixed-species planktonic culture, containing equal proportions (~10^7^ cells/mL) of each bacterial suspension; **(C)** q-PCR amplification efficiency, measured by the efficiency of each set of primers designed for specific detection of *P*. *aeruginosa* (PA_16S), *I*. *limosus* (IL_16S) or *D*. *pigrum* (DP_16S). Amplification efficiency (**E**), expressed as percentage, was determined from the slope of the standard curve plotting the log of the initial template copy number *vs C*_*t*_ (for each set of primers, the standard curves were repeated at least, twice, and the means ± SDs of the obtained amplification efficiencies are illustrated); **(D)** Experimental specificity test determined for each set of primers specifically designed for each species. Primer specificity was monitored by the outcome of the presence or absence of a specific 16S band visualized in a 1% (w/v) agarose gel. (+) indicates presence of band; (−) is indicative of absence of band. Abbreviations: PA = *P*. *aeruginosa*, IL = *I*. *limosus*, DP = *D*. *pigrum*.

Prior to biofilm quantification experiments, the quantification methods were optimized and validated to specifically detect and differentiate *P*. *aeruginosa*, *I*. *limosus* and *D*. *pigrum* in mixed-species planktonic populations (Fig. 2). In an earlier report^47^, we have already validated a multiplex PNA-FISH assay, where *P*. *aeruginosa* and *I*. *limosus* could be easily detected under fluorescence microscopy, by using specific designed PNA probes (Paer565 and Ilim569, respectively). We have also successfully applied the PNA-FISH assay combined with non-specific dye DAPI staining to visualize and discriminate *P*. *aeruginosa*, *I*. *limosus* and *D*. *pigrum* in triple-species biofilms.

For the plate count assay, a mixed-species culture containing equal proportions (~10^7^ CFU/mL) of the three bacterial suspensions was monitored onto unspecific (TSA) and specific (PIA and supplemented BCSA) solid growth media after 24 to 48 h incubation at 37 °C (Fig. 2A,B).

Based on colony morphology, it was possible to distinguish *P*. *aeruginosa*, *I*. *limosus* and *D*. *pigrum* onto unspecific TSA culture medium (Fig. 2A, left image). While not recommended in CF clinical microbiology^11^, PIA showed to be highly specific for *P*. *aeruginosa* isolation, enhancing the blue-green pyocyanin pigment produced by this strain (Fig. 2A, centred image). Likewise, the specific recovery of *I*. *limosus* could also be improved by using BCSA (a selective medium strongly recommended for *B*. *cepacia* complex) supplemented with polymyxin B and ticarcillin^48^, allowing the detection of slimy whitish *I*. *limosus* colonies after extended incubation time (Fig. 2A, right image). CFU counts (Fig. 2B) estimated either on TSA or on selective media approximated the initial concentration combined for each bacterial suspension in the mixed-species liquid culture. To the authors’ knowledge, no specific culture medium has been reported to isolate the CF less common species *D*. *pigrum*. In this study, *D*. *pigrum* counts in mixed-species cultures were estimated indirectly by the difference between TSA counts and those enumerated by selective growth media.

Because q-PCR efficiency is highly dependent on the primers used, experimental validation of the q-PCR method was firstly evaluated by measuring the amplification efficiency for each set of primers designed to specifically target the 16S rRNA gene of P. *aeruginosa*, *I*. *limosus and D*. *pigrum* species (Fig. 2C). Primers efficiency was determined by the dilution method as well as performing a temperature gradient reaction from 50 to 65 °C, in order to determine the optimal primer annealing temperature. In general, the generated standard curves showed a linear fit with slopes between approximately −3.52 (obtained for the gene targeting *I*. *limosus*: IL_16S) and −3.65 (for the gene targeting *D*. *pigrum*: DP_16S), which are equivalent to 91 to 101% of reaction efficiency and are, therefore, within the acceptable range (90–110%)^49^. The optimal annealing temperature was shown to be 58 °C, with all set of primers showing high and comparable amplification efficiencies. To prevent cross-reactivity with the remaining species in the consortia, the specificity of each set of primers designed for P. *aeruginosa*, *I*. *limosus and D*. *pigrum* was monitored by the outcome of the presence or absence of a specific 16S band visualized in a 1% (w/v) agarose gel (Fig. 2D). Experimental evidence showed high specificity for all pair of primers, with PA_16S, IL_16S and DP_16S displaying only positive result for the presence of a specific 16S band for P. *aeruginosa*, *I*. *limosus and D*. *pigrum*, respectively. Also, the presence of primer dimer or reagent contamination was not detected by the negative control (data not shown).

### Biofilm quantification by plate count, PNA-FISH and q-PCR

**Figure 3:**
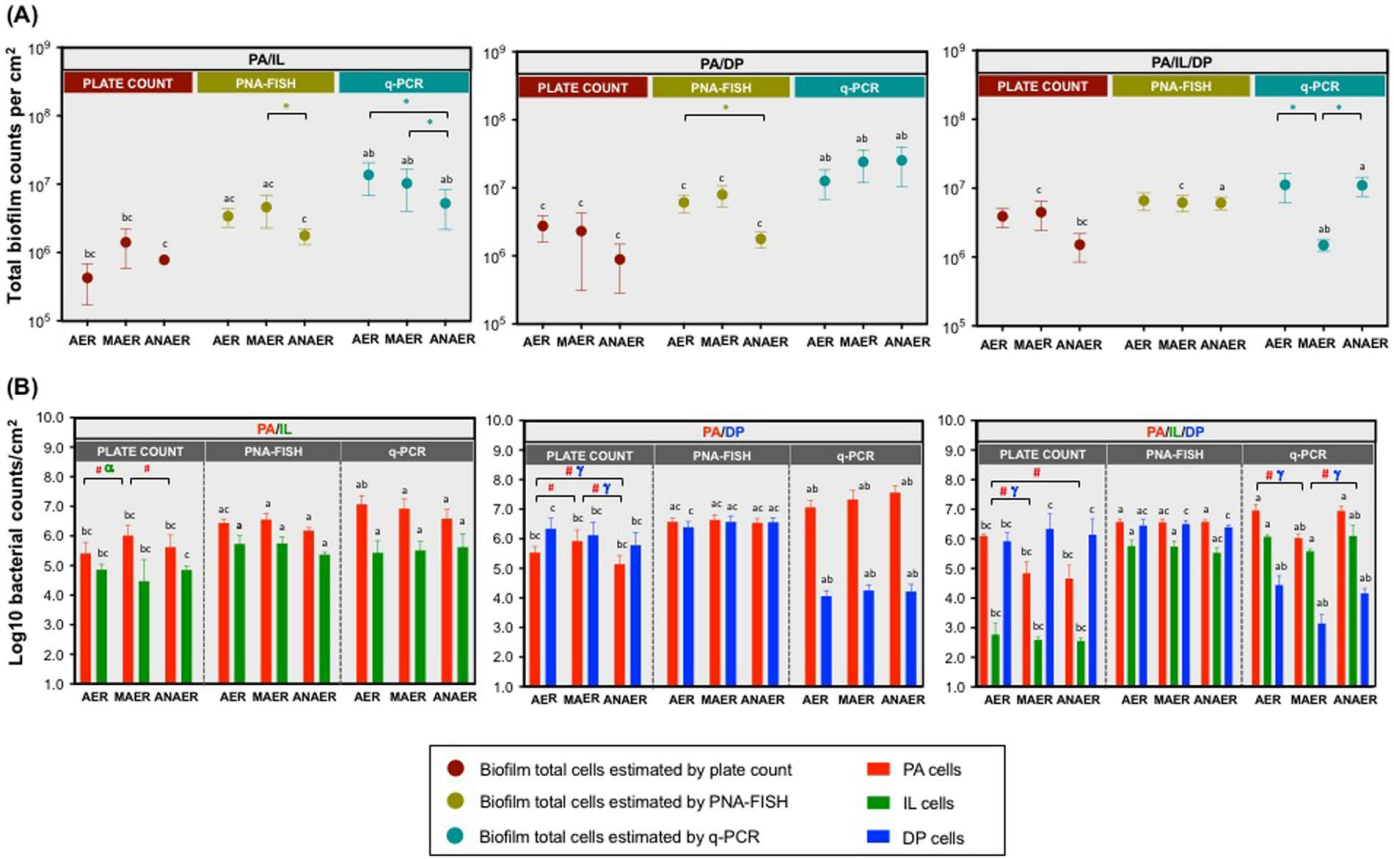
Biofilm quantification by plate count, PNA-FISH and q-PCR. (**A**) Total counts, expressed as CFU per cm^2^, estimated for biofilms formed by *P*. *aeruginosa*, *I*. *limosus* and *D*. *pigrum* developed under aerobic, microaerophilic and anaerobic conditions; (**B**) Populations of *P*. *aeruginosa*, *I*. *limosus* and *D*. *pigrum* in dual- and triple-species biofilms, estimated by the different methods. For each biofilm/condition, means ± SDs for bacterial counts are illustrated (for plate counts, three independent experiments were performed: 18 ≤ n ≤ 24; for PNA-FISH, two to three independent experiments were performed: 10 ≤ n ≤ 30; for qPCR, two to four independent experiments were performed: 6 ≤ n ≤ 9). Significant differences (*P* < 0.05) are indicated as follows: (^*^) significantly different total counts between oxygen environments; (**#)** significantly different *P*. *aeruginosa* counts between oxygen environments; (**α**) significantly different *I*. *limosus* counts between oxygen environments; **(γ)** significantly different *D*. *pigrum* counts between oxygen environments; (**a**) significantly different from plate counts; **(b**) significantly different from PNA-FISH counts; (**c**) significantly different from q-PCR counts. Abbreviations: PA = *P*. *aeruginosa*, IL = *I*. *limosus*, DP = *D*. *pigrum*, AER = aerobic, MAER = microaerophilic, ANAER = anaerobic.

Following experimental validations of culture and molecular techniques, dual- and triple-species biofilms were grown under AER, MAER and ANAER conditions and then quantified (Fig. 3). Considering the range of factors generated for this study (involving consortia with different number and physiologically distinct species; environments with distinct oxygen availabilities), one might expect *a priori* that such multifactorial heterogeneity may lead to variable outcomes in biofilm quantification by the different methods.

Total biofilm counts, as assessed by the different methods (Fig. 3A), were obtained by adding the counts for each individual population (displayed in Fig. 3B). As shown, total counts for the 6 h-old biofilms ranged between 10^5^ and 10^7^ CFU/cm^2^ of magnitude (Fig. 3A), with PNA-FISH and q-PCR displaying the highest biofilm counts. Some divergences (<1 log_10_ counts per cm^2^) could be observed between plate count and PNA-FISH. Major differences (*P* < 0.0001) were mostly noticed when culture or PNA-FISH counts were compared with q-PCR for the same oxygen environment. In addition, certain differences among counts of biofilms developed under distinct oxygen environments were also noticeable.

These disparities in biofilm counts are still mirrored at the species level (Fig. 3B) and become more pronounced when examining the triple-species consortia. For most situations, *P*. *aeruginosa* and *I*. *limosus* showed the highest counts when assessed by molecular approaches. *I*. *limosus* plate counts were significantly reduced (~ 2.5 log_10_ per cm^2^) when compared with PNA-FISH or q-PCR counts (P < 0.0001), which enumerate *I*. *limosus* populations with a difference of up to 4 log cells per cm^2^. Similar results were achieved for *P*. *aeruginosa*, that tends to reduce gradually their culture counts along with the decrease of oxygen in the environment, but still being detected in significantly greater numbers by PNA-FISH and q-PCR. These outcomes underline the notion that using exclusively a single method may not provide detailed/true insight into the biofilms composition and even render for misleading outcomes when elucidating the microbial interactions within the consortia. Intriguingly, *D*. *pigrum* counts estimated by q-PCR were markedly affected in comparison with respective quantification by PNA-FISH or culture (*P* < 0.0001 for most cases). This inconsistency in quantification was noticed for both dual- (*P*. *aeruginosa*/*D*. *pigrum*) and triple-species biofilms (*P*. *aeruginosa*/*I*. *limosus*/*D*. *pigrum*). While culture and PNA-FISH methods allowed to assess *D*. *pigrum* in great abundance (approximating 6 log_10_ cells per cm^2^) in biofilms grown under all oxygen environments (some examples of PNA-FISH images are in Supplementary Fig. S1), q-PCR estimates *D*. *pigrum* between 3 to 4 log_10_ counts per cm^2^ (*P* < 0.0001). Despite showing significant differences in individual population counts, both culture and molecular techniques have ascertained for *P*. *aeruginosa* the highest absolute numbers in the consortia, which was observed for most cases.

In brief, our findings highlight large discrepancies between culture and molecular methods in detecting and quantifying cells encased in biofilms. As we cannot precisely define the molecular mechanisms that are beyond such incongruities, it is conceivable to suggest that the external environmental conditions may have induced changes in the dynamics (e.g. microbial composition, function, metabolic processes) of the communities, with the level of complexity/heterogeneity generated inside the biofilms ultimately having potential implication in the reliability and effectiveness of the different methods (e.g. at gene expression and amplification levels in q-PCR method; at limited PNA probe diffusion into the consortium; at limiting growth on solid medium). For instance, we could notice that biofilms formed under AER, MAER and ANAER conditions showed apparently great *D*. *pigrum* abundance, which was ascertained by plate count and PNA-FISH. We may speculate that the disproportionate amount of *D*. *pigrum* cells in the consortia may be associated to a limited effectiveness of DNA extraction step and thus presenting a limited yield by q-PCR. The process of DNA extraction is often deemed as a critical step, as cell lysis efficiency might not be equal for all the microbial cells in a sample. DNA recovery differs both from the bacterial species and from the type of the sample^50,51^. *D*. *pigrum* is a Gram-positive bacterium, arranged in pairs, tetrads and/or clusters^52,53^, which can make cell disruption arduous and consequently impair nucleic acid release. After testing different approaches (including several commercially available kits) attempting to extract DNA from planktonic cultures, acceptable DNA yields (in terms of concentration and purity) were only achieved after a cell lysing pre-treatment combining chemical and mechanical lysis (a procedure generally used to lyse yeast cells). But we believed that DP DNA extraction could be likely compromised when transposed directly to biofilms. In order to confirm our theory, monospecies biofilm counts were estimated by different techniques (culture, PNA-FISH, q-PCR and DAPI), with *D*. *pigrum* counts being drastically affected by q-PCR (where the reduction achieved, in average, 3.8 log_10_ cells per cm^2^) when compared with culture or DAPI enumerations (P < 0.0001) (see Supplementary Fig. S2).

As of yet, no molecular tests suitable for comprehensive analysis of individual populations in biofilms are completely exploited (although potentially suitable technologies and instrumentation already exist), assay development and validation would have to be undertaken. Our results prove that validating a method for planktonic microorganisms is not sufficient if the final application is in biofilms. It is hard, however, to conceive a method that can be fully reliable for all physiological states that cells can be found in biofilms. The problematic here is that validation would have to be performed for every specific situation of biofilm assessment (including time, conditions, etc.), which is time-consuming and potentially unpractical. A brief appreciation on biofilm quantification studies suggests that most do not refer *a priori* calibration and/or validation of the methods used for or include optimization just for cells in suspension^13,26,34,36–38,54–57^.

### Quantification of biofilms following antibiotic treatment

**Figure 4:**
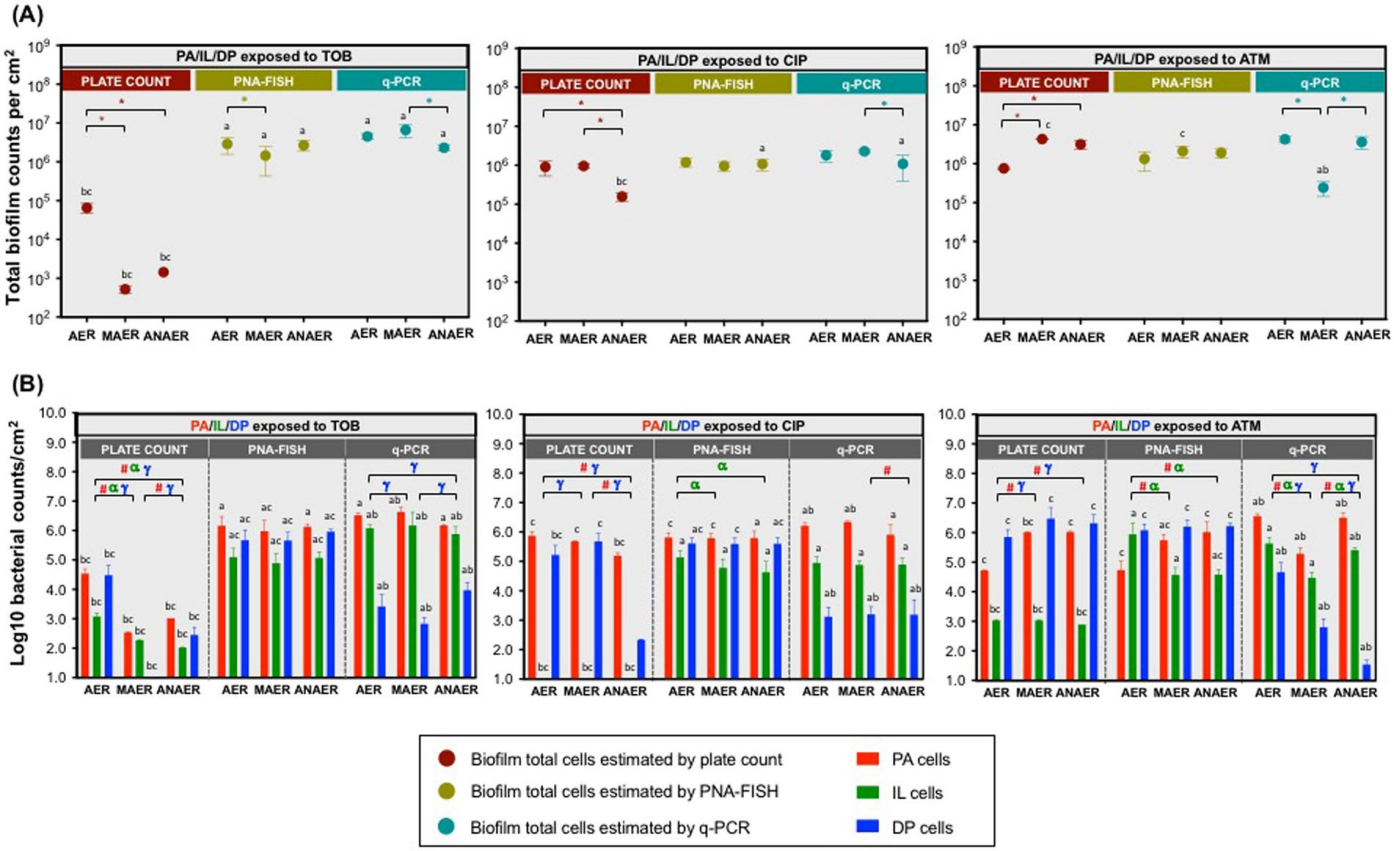
Biofilm quantification following antibiotic treatment. (**A**) Total counts, expressed as CFU per cm^2^ and (**B**) Populations of *P*. *aeruginosa*, *I*. *limosus* and *D*. *pigrum* within the defined triple-species biofilms (PA/IL/DP) exposed to 128 mg/L tobramycin, 2 mg/L ciprofloxacin and to 2 mg/L aztreonam under aerobic, microaerophilic and anaerobic conditions for 24 h. For each biofilm/condition, means ± SDs for bacterial counts are illustrated (for plate counts, three independent experiments were performed: 18 ≤ n ≤ 24; for PNA-FISH, two to three independent experiments were performed:10 ≤ n ≤ 30; for qPCR, two to four independent experiments were performed: 6 ≤ n ≤ 9). Significant differences (*P* < 0.05) are indicated as follows: (*) significantly different total counts between oxygen environments; (**#)** significantly different *P*. *aeruginosa* counts between oxygen environments; (**α**) significantly different *I*. *limosus* counts between oxygen environments; **(γ)** significantly different *D*. *pigrum* counts between oxygen environments; **(a)** significantly different from plate counts; **(b)** significantly different from PNA-FISH counts; **(c)** significantly different from q-PCR counts. Abbreviations: PA = *P*. *aeruginosa*, IL = *I*. *limosus*, DP = *D*. *pigrum*, AER = aerobic, MAER = microaerophilic, ANAER = anaerobic, TOB = tobramycin, CIP = ciprofloxacin, ATM = aztreonam.

Following biofilm development under the different oxygen environments, the next stage was to appraise for the biofilm changes promoted by the antibiotic treatment. Biofilms involving the three species were exposed for additional 24 h to sub-MBEC doses of three CF relevant antibiotics: TOB (at 128 mg/L), CIP (2 mg/L) and ATM (2 mg/L) and further quantified by means of culture-dependent and –independent methods (Fig. 4).

Overall, total cells ascertained by the different methods remained relatively constant for most biofilms before (Fig. 3A) and after antibiotic treatment (Fig. 4A). These findings are in agreement with previous studies, that showed minimal changes in bacterial counts following antibiotic treatment^12,58,59^, therefore illustrating that the overall bacterial burden remains unchanged despite treatment. Similar to that observed for untreated biofilms, the discrepancies among culture, PNA-FISH and q-PCR counts after antibiotic treatment were more notorious while assessing the individual populations (Fig. 4B). To focus on the differences in individual counts ascertained by the different methods, see Supplementary Fig. S3.

It was noticeable that the highest bacterial numbers were detected by PNA-FISH and/or by q-PCR (Fig. 4B). Similar findings were already observed for untreated biofilms. Biofilm composition showed to be highly variable, with fluctuations in population sizes being dependent on the antibiotic used. An increasing body of evidence has highlighted the effect of antibiotic treatment in the composition of the communities^40,44,60^, with recent reports even showing changes on other members of the airways microbiota beyond *P*. *aeruginosa* exerted by CIP and TOB therapies^61^. In this study, however, our surprise lies on the disparities in individual population‘ abundances relying on the method used to characterize the individual populations. And if these incongruences are well notorious in our restricted biofilm model size (of only three bacterial species), we can even speculate for a more complicated scenario when assessing a broader community, which truly reflects most polymicrobial infections. In brief, we particularly discerned a sharp decline in *I*. *limosus* quantification by culture after antibiotic intervention (even displaying negative culture in CIP-exposed biofilms). However, this decay was not observed when quantitated by molecular tools, with both still detecting *I*. *limosus* in significantly higher numbers than culture (Fig. 4B). Once again, these findings strictly reflect that culture techniques solely may not prove to be the most reliable method to undertake biofilm composition analysis, particular upon antibiotic stress, with studies identifying either bacteria that can enter a VBNC state^62,63^ or that are below the detection limit of culture^12^. In addition, for the case of fastidious and so potentially difficult to culture organisms, as is *I*. *limosus*, cultivating these species upon antibiotic pressure can be even more problematic.

Analogously to what was observed for untreated biofilms, q-PCR counts obtained for *D*. *pigrum* populations were still significantly disturbed in comparison with counts from culture and PNA-FISH (*P* < 0.0001 for most cases) which, as has been suggested, is likely related to the ineffectiveness of DNA extraction in *D*. *pigrum* biofilms.

Irrespective to the technique used to characterize the consortia, *P*. *aeruginosa* was apparently the most dominant population ascertained by all methods, a finding also observed in the case of untreated biofilms. These results suggest that, although showing to be inconsistent in giving absolute bacterial counts in biofilms, these very different methods can be employed, with some level of confidence, to detect abundant organisms in a polymicrobial community. As the most prominent organism detected in CF polymicrobial consortia, it is not therefore surprising that *P*. *aeruginosa* has been the major focus in CF antibiotic therapy^64–66^. However, it is not possible to preclude that less-abundant species cannot act as significant contributors for a more severe and recalcitrant infection, as it has been earlier reported for IL and DP^10,40^.

**Figure 5:**
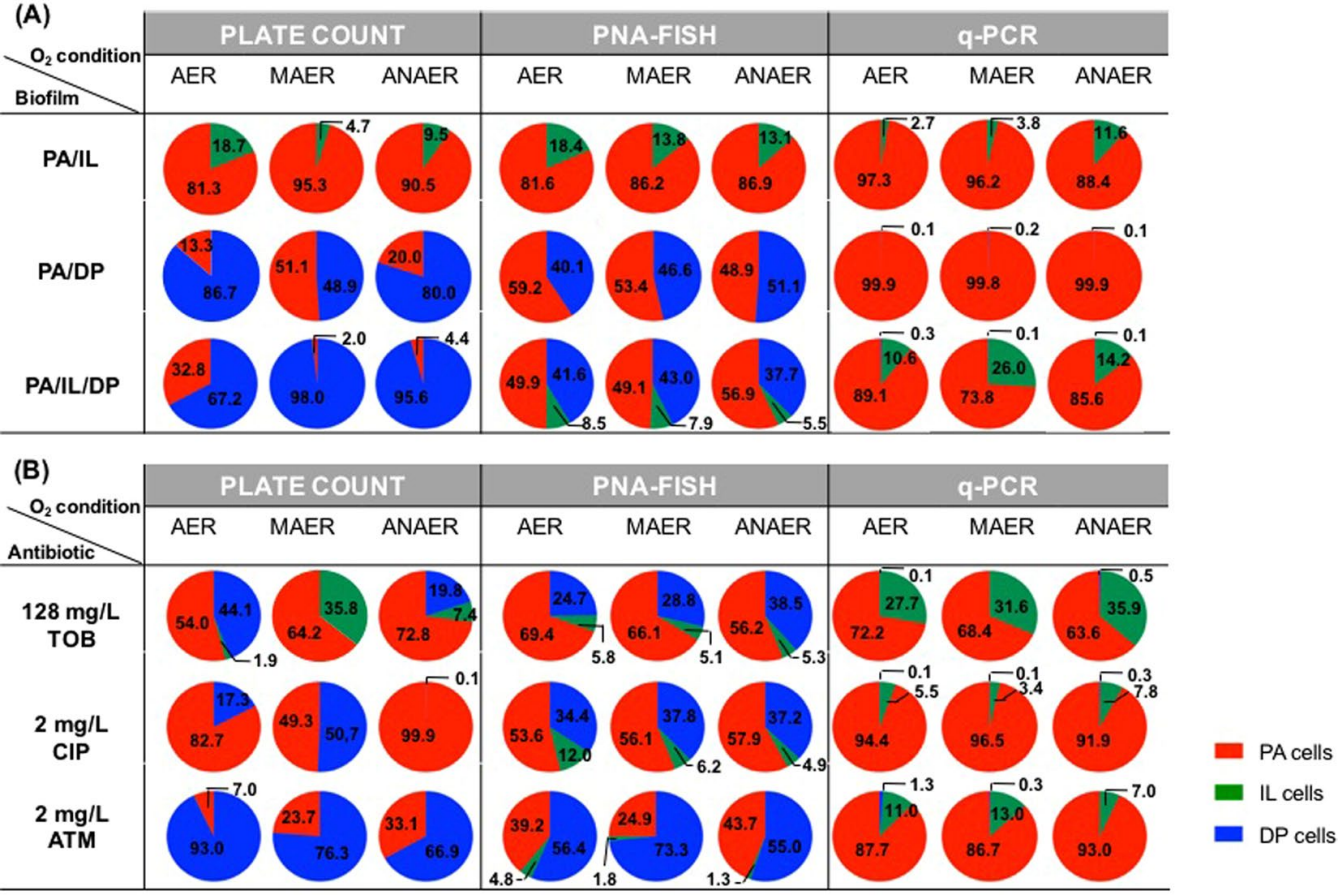
Relative distributions of the bacterial populations within biofilms, determined by culture-based and molecular methods. (**A**) Distributions for *P*. *aeruginosa*, *I*. *limosus* and *D*. *pigrum* populations in dual- and triple-species biofilms before antibiotic treatment; (**B**) and for the triple biofilms following antibiotic treatment with 128 mg/L tobramycin, 2 mg/L ciprofloxacin and 2 mg/L aztreonam, under aerobic, microaerophilic and anaerobic environments. For each condition/biofilm, means of the relative percentages of the biofilm individual populations are illustrated for two to four independent assays. Abbreviations: PA = *P*. *aeruginosa*, IL = *I*. *limosus*, DP = *D*. *pigrum*, AER = aerobic, MAER = microaerophilic, ANAER = anaerobic, TOB = tobramycin, CIP = ciprofloxacin, ATM = aztreonam.

The *P*. *aeruginosa*, *I*. *limosus* and *D*. *pigrum* abundances in biofilms assessed by the different quantification methods can be better visualized through their relative bacterial distributions in the consortia (Fig. 5). Inconsistent distributions in the biofilm individual populations were well evident while using different quantification techniques. Accordingly, a species can be determined as being the dominant/major in a biofilm sample, but only detected as a merely “contaminant” when assessed by a different quantification method. It was the case of q-PCR, which seems to select only for one species (*P*. *aeruginosa*) or two (*P*. *aeruginosa* and *I*. *limosus*) (with the relative percentage of DP in the whole consortia below 1.3%) when analysing *P*. *aeruginosa*/*D*. *pigrum* (Fig. 5A) and triple biofilms before (Fig. 5A) and following antibiotic exposure (Fig. 5B). Again, these inadequacies amongst biofilm quantification data strongly emphasises the need for a complete and rigorous calibration of the methods used for assessing the consortia.

It was noteworthy in this study that, despite the incongruities among biofilm quantification data, molecular techniques often displayed higher aptitude and sensitivity to afford greater counts for biofilm individual populations, comparing with conventional culture. In addition to q-PCR, which has been a widely used technique in analysing environmental and clinical microbiological samples, epifluorescence microscopy based methods also offer a faster and reliable alternative for monitoring polymicrobial biofilm communities. In particular, PNA-FISH effectively extends epifluorescence microscopy, allowing for a rapid discrimination, location and/or enumeration of bacterial populations in polymicrobial communities^40,47,67–74^. In our study, to make biofilm-cells counts easy and homogeneous (which likely facilitates in the case of thick and more dense biofilms), PNA-FISH assay was performed *ex situ* to quantitatively monitor bacterial populations in the multispecies consortia^69,70^.

Despite the potential of the molecular tools to circumvent some of the culture flaws, frequently providing a more rapid and sensitive assessment of the species present in polymicrobial infections, it must be recognized that these techniques can also introduce potential bias. For instance, q-PCR requires designing primer sequences for each species present in the sample. As we know which bacteria were included in our consortia, we only need to design sets of oligonucleotide primers to specifically target those species. But with the literally thousands of microbes existing in most polymicrobial infections, constructing thousands of primers for each analysis is inefficient, costly and currently impractical^75^. Furthermore, prediction need to be made as to which agent is likely to be associated with a particular infection^76^. Also, FISH presents numerous advantages when compared with culture-dependent techniques and even with q-PCR (*e*.*g*. avoiding DNA extraction or PCR processes), but has several limitations. Likewise to PCR, knowledge of the nucleotide sequence of the target organisms in the samples is needed to design new probes which, together with the optimization of the hybridization conditions makes the processes time-consuming and labor-intensive^47,77^. Also, the efficiency of the hybridization might be influenced by the physiological state of the cells^47,78^. To conclude, the bulk cost of the technique is particularly related with the commercial value of PNA probes, which are relative expensive^67^, likely supporting its limited use in routine microbial diagnosis in many clinical microbiology laboratories.

## Conclusions

The importance of examining in detail the microbial composition of biofilms is critical while we now recognize the truly polymicrobial nature of many biofilm infections, ranging from clinic (lung/respiratory tract^79^, oral cavity^80^, skin^81^, gut^82^, stomach^83^, urinary tract^84^) to environmental/industrial sources (marine sediments^85^, industrial bioreactor, sludge^86^). Providing detailed insights into the diversity and abundances of inhabiting microbes is of great importance for a better understanding on the role that polymicrobial biofilms may play in their natural context alongside with an improvement in the likelihood of treatment success.

This study observed relevant inconsistencies between conventional culture and molecular tools in detecting and quantifying bacterial populations inhabiting in a biofilm. Based on our findings, we recommend using at least two, but preferentially three methodological approaches, to analyse complex polymicrobial communities such as the ones in CF. It is envisioned that combining more than one methodological approach will be valuable to circumvent the caveats of each technique alone, tailoring a more complete picture of a broad range of polymicrobial biofilm communities, ultimately unlocking accurate microbiome-associated treatment decisions and potentially yielding novel avenues aimed at effectively controlling/eradicating detrimental polymicrobial biofilms.

## Materials and Methods

### Biofilm formation and preparation for quantification analysis

Two- and triple-species biofilms encompassing the major pathogen *P*. *aeruginosa* (UCBPP-PA14) and two less typical species - *I*. *limosus* (strain M53, isolated from CF sputum), and *D*. *pigrum* (CIP 104051 T, purchased from Institute Pasteur Collection, Paris, France) - identified in CF infections^87^, were developed for 6 h under environments with distinct oxygen availabilities - aerobic (AER), microaerophilic (MAER) and anaerobic (ANAER) - using procedures described in earlier reports^40,88^.

The triple consortia were then exposed to 128 mg/L tobramycin (TOB), 2 mg/L ciprofloxacin (CIP) and to 2 mg/L aztreonam (ATM) (all from Sigma-Aldrich, St. Louis, MO, USA). TOB, CIP and ATM are typically used as first-line treatments to control or eradicate *P*. *aeruginosa* CF pulmonary infections^65^. The selected antibiotic concentrations were sub-therapeutic (below values obtained for Minimum Biofilm Eradication Concentration, MBEC), determined elsewhere^40^ to ensure that biofilms were not completely eradicated and allowing to address changes in biofilm microbial compositions. Stock antibiotic solutions were prepared at 1000 mg/L, following the manufacturer’s instructions. After biofilm formation, the wells were rinsed with sterile distilled water to remove non-adhered cells and the triple-species biofilms were exposed to freshly working antibiotic solutions (prepared by diluting the antibiotic from stock solution in cation-adjusted Mueller-Hinton broth (CAMHB) until the desired final concentration). Plates were then incubated for 24 h at 37 °C, under AER, MAER and ANAER conditions. The experimental design and workflow of our strategy for biofilm quantification analysis is outlined in Fig. 1.

### Assessment of individual populations in biofilms

After discarding the planktonic cell fractions (supernatant) from untreated and antibiotic-treated biofilms, the wells were rinsed twice and microbial composition of biofilms was analyzed throughout culture- and molecular-based techniques:

#### Plate count (culture-based) method

After the rinsing step, wells were filled with 0.9% (w/v) saline solution (NaCl; J. T. Baker, The Netherlands) and biofilms were detached by sonication using an ultrasound bath (Sonicor, model SC-52, UK) operating at 50 kHz, during 10 min and then resuspended by pipetting up and down, as recently described^40^. After sonication, 1 mL of biofilm suspension was transferred to an Eppendorf tube and vortexed vigorously for 30 s. Serial 10-fold dilutions from each biofilm sample were made on saline solution and 10 μL drops of each dilution were plated onto TSA and on selective agar media to discriminate each bacterial population in the consortia. For PA quantification, plates containing *Pseudomonas* isolation agar (PIA; Sigma) were used. For *I. limosus* isolation, *Burkholderia cepacia* selective agar, (BCSA; Oxoid Ltd, Hampshire, UK) supplemented with 300 000 IU/L polymyxin B (Biochrom, Berlin, Germany) and 100 mg/L ticarcillin (Sigma) was employed. Estimation of *D*. *pigrum* in the dual- and triple-species biofilms was based on the difference between the average total cell number (determined by counts in TSA) and the average number of bacteria, obtained from selective culture media, presented in the consortia. To validate the culture method, a mixed-planktonic suspension containing the three bacteria combined in equal proportions (at ~10^7^ cells/mL) was monitored on TSA and on selective media.

#### PNA-FISH multiplex assay

A multiplex FISH assay using two specific PNA probes, Paer565 and Ilim569, previously developed, optimized and validated for *P*. *aeruginosa* and *I*. *limosus* detection, respectively^47^, was followed. At the end of the hybridization procedure, an additional staining step with 4′, 6-diamidino-2-phenylindole (DAPI; Sigma) was carried out to identify the third organism (*D*. *pigrum*).

PNA-FISH assay was performed *ex situ*, according with previous procedures^69,70^. Briefly, after rinsing biofilms, the wells were scraped in 1 mL of ultrapure sterile water. The resulting biofilm-cells suspensions were then pipetted up-and-down, transferred to an Eppendorf tube and vortexed for 30 s at maximum speed. Afterwards, 20 μL of each biofilm sample were spread in 8 mm well diagnostic glass slides (ThermoScientific, Braunschweig, Germany) and let to air-dried prior to fixation. The fixation, hybridization and the probes washing procedures were strictly followed as described before^47^. At the end, the smears were allowed to air dry, immersed in 20 μL of DAPI (40 μg/mL) for 10 min and incubated at room temperature in the dark. Prior to microscopy, samples were mounted with one drop of non-fluorescent immersion oil (Olympus, Tokyo, Japan) and analysed using an epifluorescence microscope (Olympus BX51) coupled with a DP72 digital camera and three sets of filters (DAPI – 360-370/420; FITC – 470–490/520, sensitive to detect Alexa Fluor 488 molecule attached to the Ilim569 probe; and TRITC – 530–550/590, sensitive to detect Alexa Fluor 594 molecule attached to the Paer565 probe) (Olympus Portugal SA, Porto, Portugal). All images were acquired using the Olympus cellSens software. A total of 10 to 30 fields with an area of 5671,3 μm^2^ were counted and the average was used to estimate the *P*. *aeruginosa*, *I*. *limosus* and *D*. *pigrum* cells per cm^2^. At least, two independent experiments were performed for each condition.

#### Quantitative Real-time PCR (q-PCR) assay

First, 20–22 bp oligonucleotide primers for the detection of 16S rRNA reference genes were designed using the Primer3 web-based software (http://frodo.wi.mit.edu/)^89^, having *P*. *aeruginosa* PAO1 (PubMed accession number NC_74828.1), *I*. *limosus* M53 (PubMed accession number JF803524.1) and *D*. *pigrum* NCFB 2975 (PubMed accession number X70907.1) genome as templates (Table 1). Potential candidates for PCR primers were compared to the aligned SSU-rRNA database of the Ribosomal Database Project II (RDP-II) using the PROBE MATCH utility^90^ and were compared to all available 16S rRNA sequences by using the BLAST database search program (http://blast.ncbi.nlm.nih.gov/Blast.cgi)^90^.

**Table 1:** Sequences of oligonucleotide primers to target 16S rRNA reference genes used in this work.

Primer	Sequence (5′−3′)	Target species	Location^a^	Product size (bp)
PA_16 S_FW	CTCAGACACAGGTGCTGCAT	*P*. *aeruginosa*	1031–1050	130
PA_16S_RV	CACCGGCAGTCTCCTTAGAG		1141–1160	
IL_16S_FW	CGACGATGATGACGGTAGTG	*I*. *limosus*	341–360	167
IL_16S_RV	AATGCAGTTCCCAGGTTGAG		488–507	
DP_16S_FW	TGATTGATTAGTGGCGAACG	*D*. *pigrum*	77–96	221
DP_16S_RV	CACCCTCTCAAGTCGGCTAC		278–297	

^a^Position and size relative to 16S rRNA sequence of the template organisms.

### Assessment of primers efficiency and specificity

The q-PCR amplification efficiency was obtained by measuring the efficiency of each primer pair, which was determined through the dilution method and established by means of a calibration curve^91^. Briefly, for each species, serial dilutions from a genomic DNA (gDNA) sample of known concentration (50 ng/μL) were made in RNAse free water and amplification of standard dilution series was followed by performing as described in the sections below, under temperature gradient reaction ranging from 50 to 65 °C. The standard curve was generated by plotting the log of the initial template copy number against the cycle threshold (*C*_*t*_, i.e. the threshold cycles in which exponential amplification of PCR products was first detected) generated for each dilution. Amplification efficiency (E), expressed as percentage, was then determined from the slope of the log-linear portion of the calibration curve, as follows: E = 10^−1/slope^ − 1. At least 4 points were used to construct each curve and each gDNA concentration was run in duplicate. Because at 58 °C, all set of primers had the best and more similar efficiencies value, this annealing temperature was used in the q-PCR protocol for further assays. The specificity of each set of primers was evaluated by visualizing the presence or absence of specific 16S band in a 1% (w/v) agarose gel (electrophoresis was carried-out at 90 V for 45 min) for each species and also observing for non-specific binding (e.g. primer dimer formation). Furthermore, a melting curve analysis was performed for all primer sets, in all experiments, to ensure a single peak, indicative of primer specificity.

### Extraction of genomic DNA from biofilms

In order to extract gDNA from biofilms, 24 wells from 24-well plates were scraped per condition and the biofilm disrupted-cells (concentrated in 3 mL sterile distilled water) were collected in 15 mL polypropylene conical tubes. To remove extracellular DNA, the biofilm matrix was extracted by sonicating the biofilm samples in ice in an ultrasonic processor (Cole-Parmer, IL, USA) for 30 s at 30% amplitude, followed by a vortexing step of 30 s and centrifugation (5000 × g; 10 min)^92^. To ensure that sonication do not interfere with cell cultivability, CFU from biofilm samples were confirmed after sonication (data not shown). After discarding the supernatant, the biofilm-cell pellet was resuspended in 200 μL of lysis buffer (containing 2% (v/v) triton X-100 (at pH 10; Sigma-Aldrich, St. Louis, MO, USA), 1% (w/v) sodium dodecyl sulphate (Sigma), 100 mM NaCl, 10 mM Tris-HCl (at pH 8.0; Sigma) and 1 mM disodium EDTA (Sigma) and transferred to a sterile Eppendorf tube containing 0.3 g acid-washed glass beads (150–212 μm diameter) (Sigma). Afterwards, 200 μL of phenol:chloroform:isoamyl alcohol 25:24:1 (Sigma) were added and the samples vortexed vigorously for 3 min. Then, 200 μL of TE Buffer (containing 10 mM Tris-HCl at pH 8.0 and 1 mM disodium EDTA) were added and centrifuged (12000 × g; 5 min). The aqueous phase was transferred to a new DNAse/RNAse-free Eppendorf tube and after adding 1 mL of absolute ethanol (Fisher Scientific, Leicester, UK) (and mixed by inversion), the gDNA was allowed to precipitate by incubating the samples at −20 °C (overnight). Afterwards, the samples were centrifuged (12000 × g; 3 min) and the pellet was resuspended in 400 μL of TE Buffer. Then, 30 μL of RNAse A (at 1 mg/mL) were added, and the samples were incubated for 5 min at 37 °C, to remove any RNA contaminants. After incubation, 10 μL of 3 M sodium acetate and 1 mL absolute ethanol were added. The samples were mixed by inverting the tubes and centrifuged for 23 min (12000 × g). Lastly, the supernatant was discarded and the pellet was allowed to air-dry, before resuspended it in 30 μL with RNAse free water (Cleaver Scientific Ltd, Warwickshire, UK). The concentration and purity of the total gDNA was spectrometrically assessed using a NanoDrop 1000™ (Thermo Scientific, Waltham, MA, US). The absorbance ratios A_260_/A_280_ were used as indicators of protein contamination and A_260_/A_230_ as indicators of polysaccharide, phenol, and/or chaotropic salts contamination^93^. The integrity of the total gDNA was assessed by visualization of the 16S banding pattern in a 1% (w/v) agarose gel. Total gDNA extractions were performed two to four times and samples were kept at −20 °C before amplification.

### Biofilm DNA amplification in q-PCR

The amplification reactions were carried out in a total volume of 10 μL, which consisted of 2 μL of DNA samples and 8 μL of the master mixture. The latter contained 500 nM of each primer, 5 μL of 2 × of the commercial q-PCR master mix SsoFast**™** EvaGreen**®** Supermix (Bio-Rad, Hercules, CA, USA). Amplifications were performed on a CFX96^TM^ thermal cycler (Bio-Rad), comprising 1 cycle of 2 min at 98 °C (hot start) followed by 40 cycles of 98 °C for 5 s for denaturation. Annealing step was performed for 5 s at 58 °C and extension was made at 65 °C to 95 °C for 5 s (with temperature increments of 1 °C). The q-PCR products were analyzed by melting curves for unspecific products or primer dimer formation. Fluorescence was measured after each cycle. Each assay was carried out in duplicate and the average *C*_*t*_ value from each duplicate was used for analysis.

### Quantification by q-PCR

To quantify the individual populations in mixed species biofilms, standard curves were constructed for pure cultures of *P*. *aeruginosa*, *I*. *limosus* and *D*. *pigrum*. Basically, several colonies of fresh subcultures of each organism were collected and suspended in TSB. Serial 10-fold dilutions were made from each culture and dilution samples were plated onto TSA and plates incubated at 37 °C for 24–48 h. Colonies were counted in order to calculate the number of CFUs per dilution tube. The average of three replicates was determined and data was presented as CFU per mL. One milliliter from each dilution tube was subjected to gDNA extraction as described above, and samples were further analyzed concomitantly by q-PCR. For the construction of standard curves for each of the three bacteria, the *C*_*t*_ values were plotted against CFU number from the corresponding dilution sample through a tendency line. The standard curves were used to determine the CFU numbers within biofilms by converting *C*_*t*_ values obtained for consortia to CFU numbers by the tendency line equation for each organism. This approach was used because for biofilms it is easier to understand results in actual CFU numbers than in DNA concentration or copy numbers. Additionally, results are easily compared with those obtained by culture.

### Differences in bacterial counts ascertained by culture and molecular methods

To measure the variations in *P*. *aeruginosa*, *I*. *limosus* and *D*. *pigrum* cell numbers within biofilms ascertained by culture and molecular methods, ΔLog_10_ cells/cm^2^ was defined as the difference between the number of cells detected by assay A and by assay B, respectively:

$${\rm{\Delta }}{\mathrm{Log}}_{10}\,{\rm{cells}}/{{\rm{cm}}}^{2}={{\rm{N}}}_{{\rm{a}}{\rm{s}}{\rm{s}}{\rm{a}}{\rm{y}}{\rm{A}}}-{{\rm{N}}}_{{\rm{a}}{\rm{s}}{\rm{s}}{\rm{a}}{\rm{y}}{\rm{B}}}$$

where **N** is the value of mean log_10_ CFU/cm^2^ obtained for each bacterial population in the biofilm. Therefore, ΔLog_10_ cells/cm^2^ values were determined for each species in the biofilm and for each pair of assays (Culture *vs* PNA-FISH; Culture *vs* q-PCR and PNA-FISH *vs* q-PCR), and then interpreted as follows: a ΔLog_10_ cells/cm^2^ value equal to 0 indicates no variation in bacterial counts estimated by assay A and B; a positive ΔLog_10_ cells/cm^2^ value indicates higher bacterial counts estimated by assay A; a negative ΔLog_10_ cells/cm^2^ indicates higher bacterial counts estimated by assay B.

### Statistical analysis

Graph production and data were analyzed using Prism Version 7.0a for Macintosh. Unless otherwise stated, means ± SDs are illustrated for each biofilm/condition. The number of independent assays (technical replicates) ranged between two and four, depending on the method. The number of biological replicates (n) in each independent assay was between 6 and 30, as stated in each figure legend. In general, for plate counts, three independent experiments were performed: 16 ≤ n ≤ 24; for PNA-FISH, two to three independent experiments were performed: 10 ≤ n ≤ 30; for qPCR, two to four independent experiments were performed: 6 ≤ n ≤ 9. One-way or two-way analysis of variance (ANOVA) tests were used to investigate significant differences between independent groups of data. A Tukey correction was applied to the *p* value to account for multiple comparisons of data. Differences were considered statistically significant for *P* values < 0.05.

### Data availability

The datasets generated during and/or analyzed during the current study are available from the corresponding author on reasonable request.

## Electronic supplementary material

Supplementary Information
