# Supplementary material for: Quantitative assessment of individual populations within polymicrobial biofilms
Source: Sci Rep. 2018 Jun 22;8:9494. doi: 10.1038/s41598-018-27497-9 (PMC6015014; doi:10.1038/s41598-018-27497-9)
Supplement: Supplementary file 1 — Supplementary Information [file 41598_2018_27497_MOESM1_ESM.pdf]

Quantitative assessment of individual populations within polymicrobial biofilms

Susana Patrícia Lopes, Nuno Filipe Azevedo, Maria Olívia Pereira

Supplementary Fig. S1

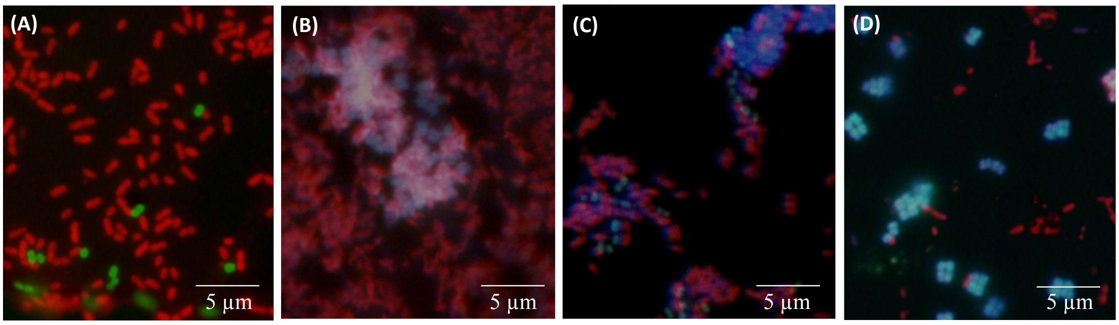

Supplementary Figure S1: Examples of PNA-FISH images used to estimate the bacterial populations in dual- and/or triple-species biofilms. The multiplex PNA-FISH assay was performed by applying the two PNA probes (Paer565 and Ilim569) and DAPI staining: **(A)** *P. aeruginosa* (red cells) and *I. limosus* (green cells) dual-species biofilms developed under aerobic conditions for 6 h; **(B)** *P. aeruginosa* and *D. pigrum* (blue stained-cells) dual-species biofilms developed under aerobic conditions for 6 h; **(C)** *P. aeruginosa*, *I. limosus* and *D. pigrum* triple-species biofilms developed under aerobic conditions for 6 h; **(D)** *P. aeruginosa*, *I. limosus* and *D. pigrum* triple-species biofilms following antibiotic treatment with 2 mg/L ciprofloxacin under aerobic environment for 24 h.

Supplementary Fig. S2

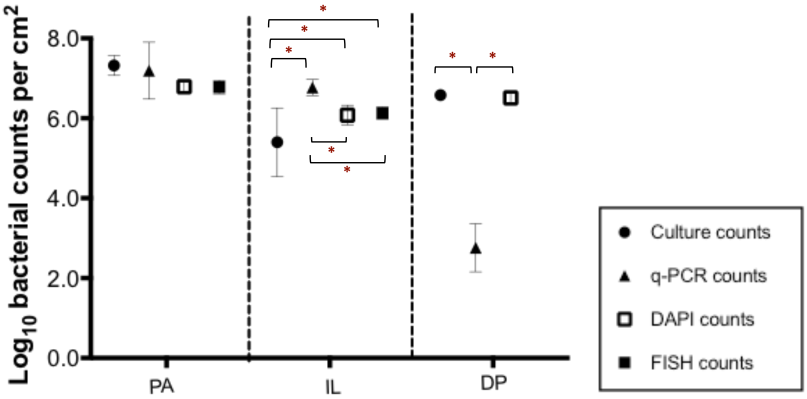

Supplementary Figure S2: Bacterial counts in single-species biofilms estimated by culture, q-PCR, DAPI and PNA-FISH. Comparison among quantification of *P. aeruginosa*, *I. limosus* and *D. pigrum* populations in single-species biofilms. *D. pigrum* counts were significantly lower when estimated by q-PCR. No PNA-FISH counts are showed for *D. pigrum*, as no probe was designed for that species. Biofilm counts are represented as means ± SDs for two to three independent experiments (culture counts: n = 16; q-PCR counts: n = 8; PNA-FISH and DAPI: 10 ≤ n ≤ 30)). *$P<0.05$ (two-way ANOVA, Tukey`s multiple comparison test) indicate significant differences between counts otained from the different methods. Abbreviations: PA = *P. aeruginosa*, IL = *I. limosus*, DP = *D. pigrum.*

Supplementary Figure S3

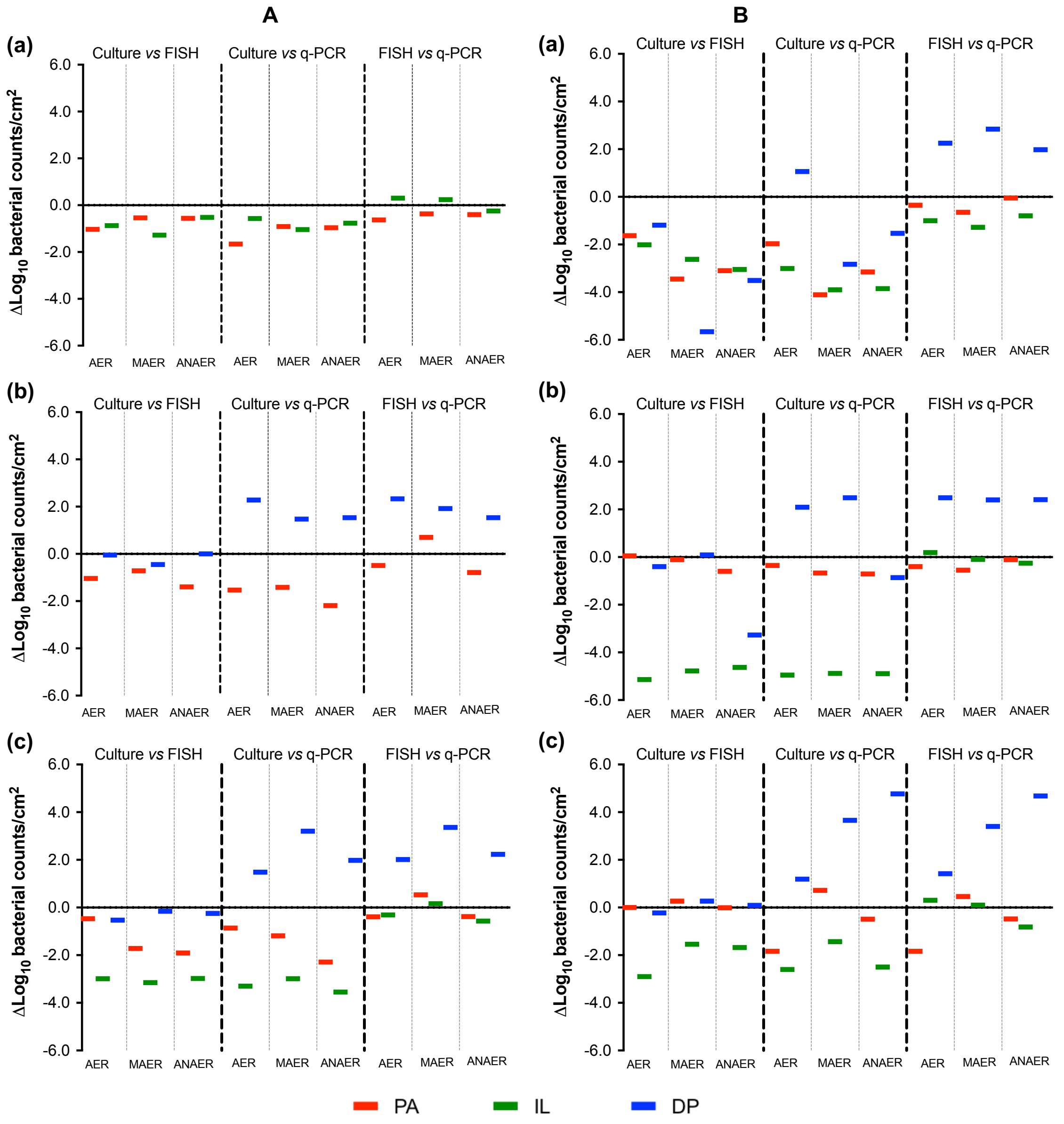

Supplementary Figure S3: Differences in bacterial counts ascertained by culture and molecular methods (q-PCR and PNA-FISH). Panel **A** represents differences in dual- and triple-species biofilms before antibiotic treatment: **(a)** *P. aeruginosa/I. limosus,* **(b)** *P. aeruginosa/D. pigrum* and **(c)** *P. aeruginosa/I. limosus/D. pigrum;* Panel **B** shows differences in the triple-species biofilms following antibiotic treatment with: **(a)** 128 mg/L tobramycin, **(b)** 2 mg/L ciprofloxacin and **(c)** 2 mg/L aztreonam, under aerobic, microaerophilic and anaerobic environments. Averages of differences in bacterial counts estimated by each pair of methods are illustrated for each species and condition. Abbreviations: PA = *P. aeruginosa*, IL = *I. limosus*, DP = *D. pigrum,* AER = aerobic, MAER = microaerophilic, ANAER = anaerobic.
